# Supplementary material for: Zika virus induces persistent phenotypic changes in natural killer cells distinct from dengue virus infection
Source: bioRxiv. 2025 Sep 16:2025.09.15.676203. Preprint. [Version 1] doi: 10.1101/2025.09.15.676203 (PMC12458331; doi:10.1101/2025.09.15.676203)
Supplement: 1 [file NIHPP2025.09.15.676203V1-supplement-1.pdf]

bioRxiv preprint doi: https://doi.org/10.1101/2025.09.15.676203; this version posted September 16, 2025. The copyright holder for this preprint (which was not certified by peer review) is the author/funder, who has granted bioRxiv a license to display the preprint in perpetuity. It is made available under aCC-BY 4.0 International license.

Supplemental information

Fig S1: CyTOF gating strategy
a) NK cells
b) PBMCs
c) Infected cells

Fig S2: MSI of all NK markers by time post-infection

Fig S3: MSI of selected NK markers by maturation & education status
a) Immature ($CD56^{bright}CD16^{-}$, cluster 8)
b) Mature ($CD57^{+}$, clusters 1, 2, and 6)
c) Uneducated (KIR^{low}, clusters 3, 7, and 8)
d) Educated (KIR^{high}, clusters 1, 2, 6, and 9)
e) Adaptive ($NKG2C^{high}$, cluster 1)

Fig S4: Unsupervised clustering of PBMCs
a) Heatmap of median protein expression and
b) Cluster frequencies of original parc clustering (resolution = 0.12) before merging of cluster 1 and 5 to CD4 T cells and clusters 2 and 4 for CD8 T cell clusters
c) Re-clustering of cells in original cluster 8 with resolution 0.12

Fig S5: CytoGLM of PBMC subsets
a) Healthy (n = 13-14) vs. Post-acute (n = 9-10)
b) Acute vs. Post-acute (n = 7 pairs)

Fig S6: Integration of ZIKV and DENV CyTOF datasets.
a) Marker distributions by batch (Z=this study, D=McKechnie *et al* 2020 [20])
b) Mean signal intensity (MSI) of all NK cell markers after data integration

Table S1: Ligand CyTOF panel info

Table S2: NK CyTOF panel info

File S1: Differential state testing results of Acute vs. Post-acute and Post-acute vs. Healthy in PBMC clusters

Figure S1

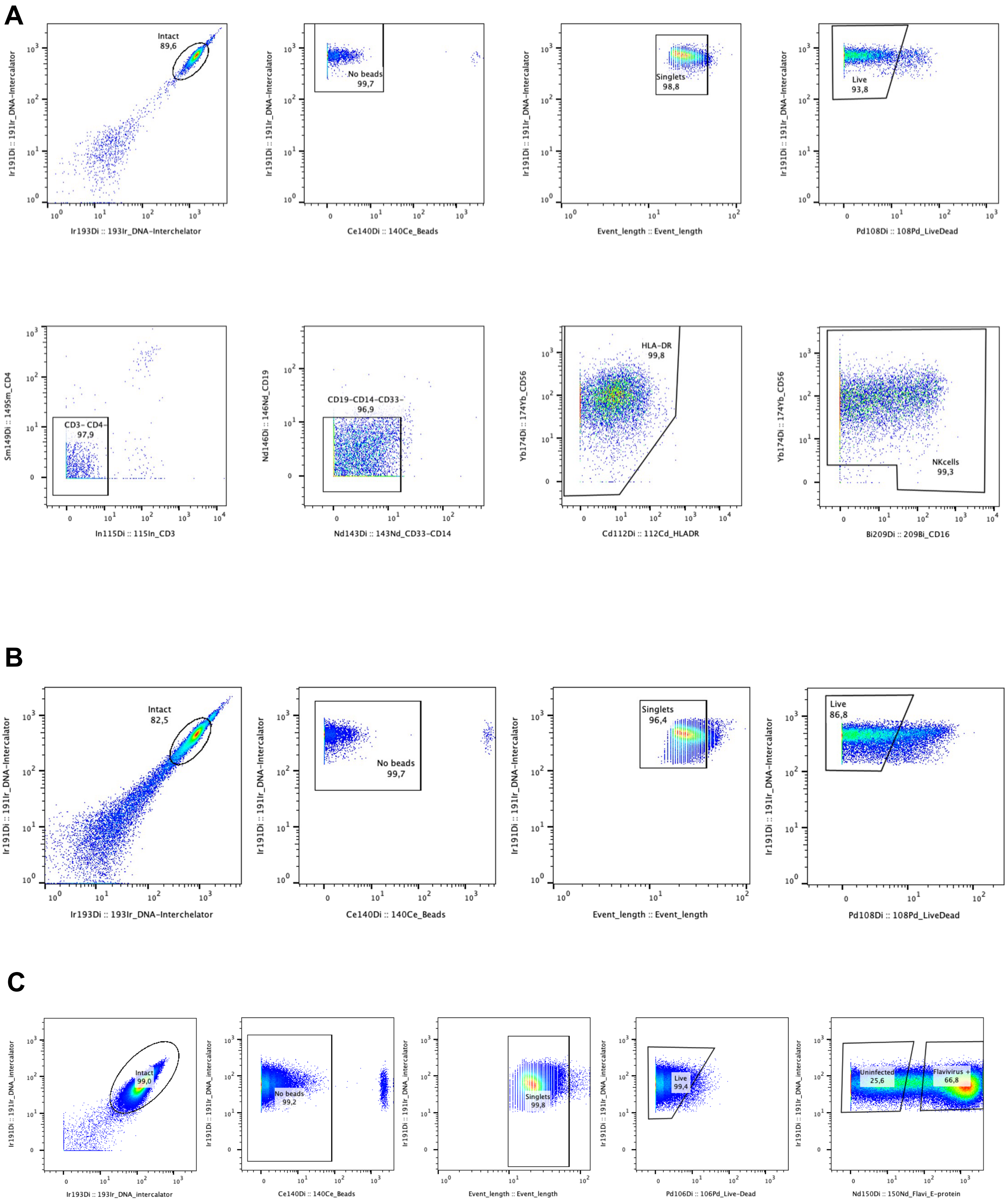

A
Intact 89,6
No beads 99,7
Singlets 98,8
Live 93,8
Ir191Di :: 191Ir_DNA-Intercalator
Ir193Di :: 193Ir_DNA-Interchelator
Ce140Di :: 140Ce_Beads
Event_length :: Event_length
Pd108Di :: 108Pd_LiveDead
CD3- CD4- 97,9
CD19-CD14-CD33- 96,9
HLA-DR 99,8
NKcells 99,3
Sm149Di :: 149Sm_CD4
In115Di :: 115In_CD3
Nd146Di :: 146Nd_CD19
Nd143Di :: 143Nd_CD33-CD14
Yb174Di :: 174Yb_CD56
Cd112Di :: 112Cd_HLADR
Bi209Di :: 209Bi_CD16
B
Intact 82,5
No beads 99,7
Singlets 96,4
Live 86,8
Ir191Di :: 191Ir_DNA-Intercalator
Ir193Di :: 193Ir_DNA-Interchelator
Ce140Di :: 140Ce_Beads
Event_length :: Event_length
Pd108Di :: 108Pd_LiveDead
C
Intact 99,0
No beads 99,2
Singlets 99,8
Live 99,4
Uninfected 25,6
Flavivirus + 66,8
Ir191Di :: 191Ir_DNA_intercalator
Ir193Di :: 193Ir_DNA_intercalator
Ce140Di :: 140Ce_Beads
Event_length :: Event_length
Pd106Di :: 106Pd_Live-Dead
Nd150Di :: 150Nd_Flavi_E-protein

Figure S2

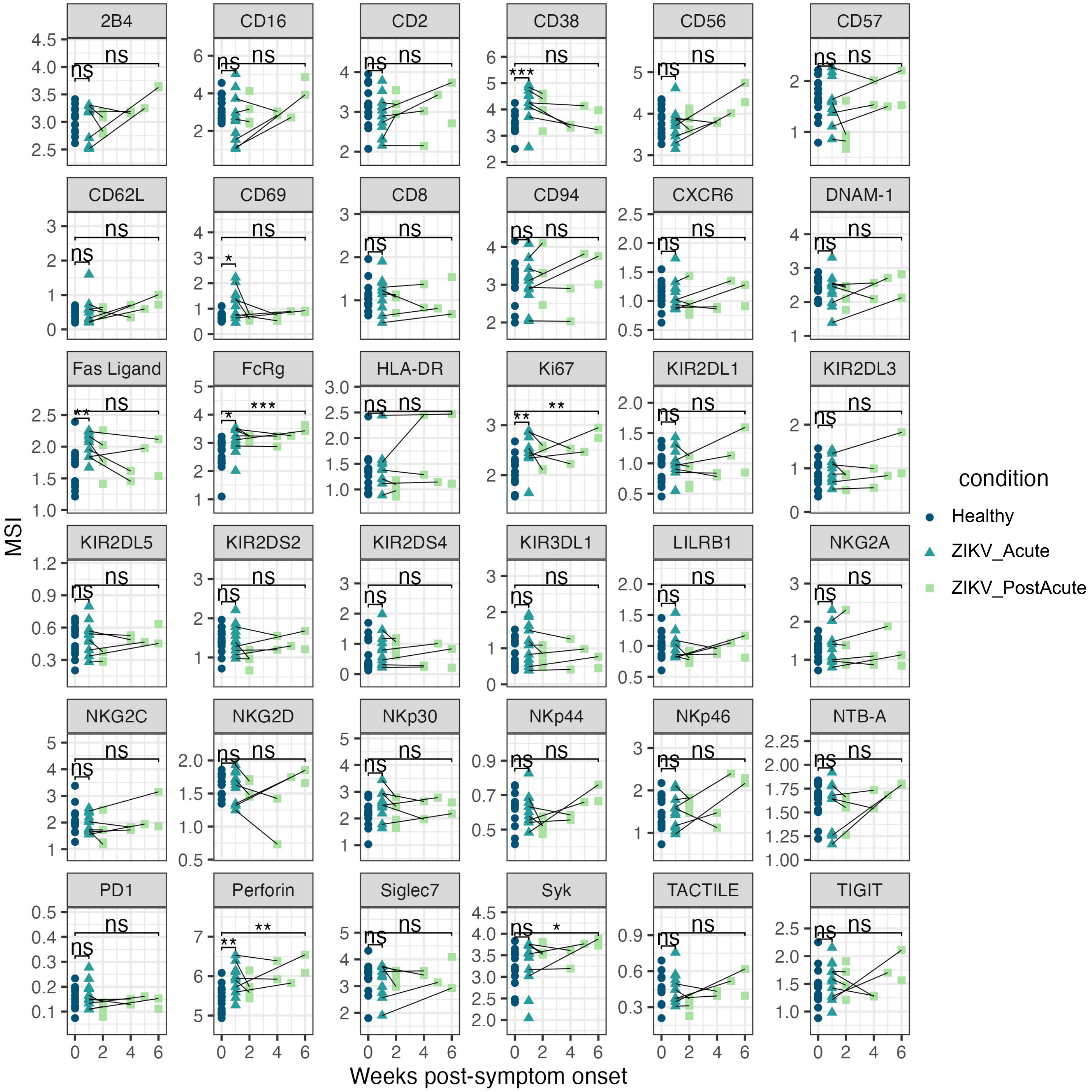
2B4
CD16
CD2
CD38
CD56
CD57
CD62L
CD69
CD8
CD94
CXCR6
DNAM-1
Fas Ligand
FcRg
HLA-DR
Ki67
KIR2DL1
KIR2DL3
KIR2DL5
KIR2DS2
KIR2DS4
KIR3DL1
LILRB1
NKG2A
NKG2C
NKG2D
NKp30
NKp44
NKp46
NTB-A
PD1
Perforin
Siglec7
Syk
TACTILE
TIGIT
MSI
Weeks post-symptom onset
condition
Healthy
ZIKV_Acute
ZIKV_PostAcute

Figure S3

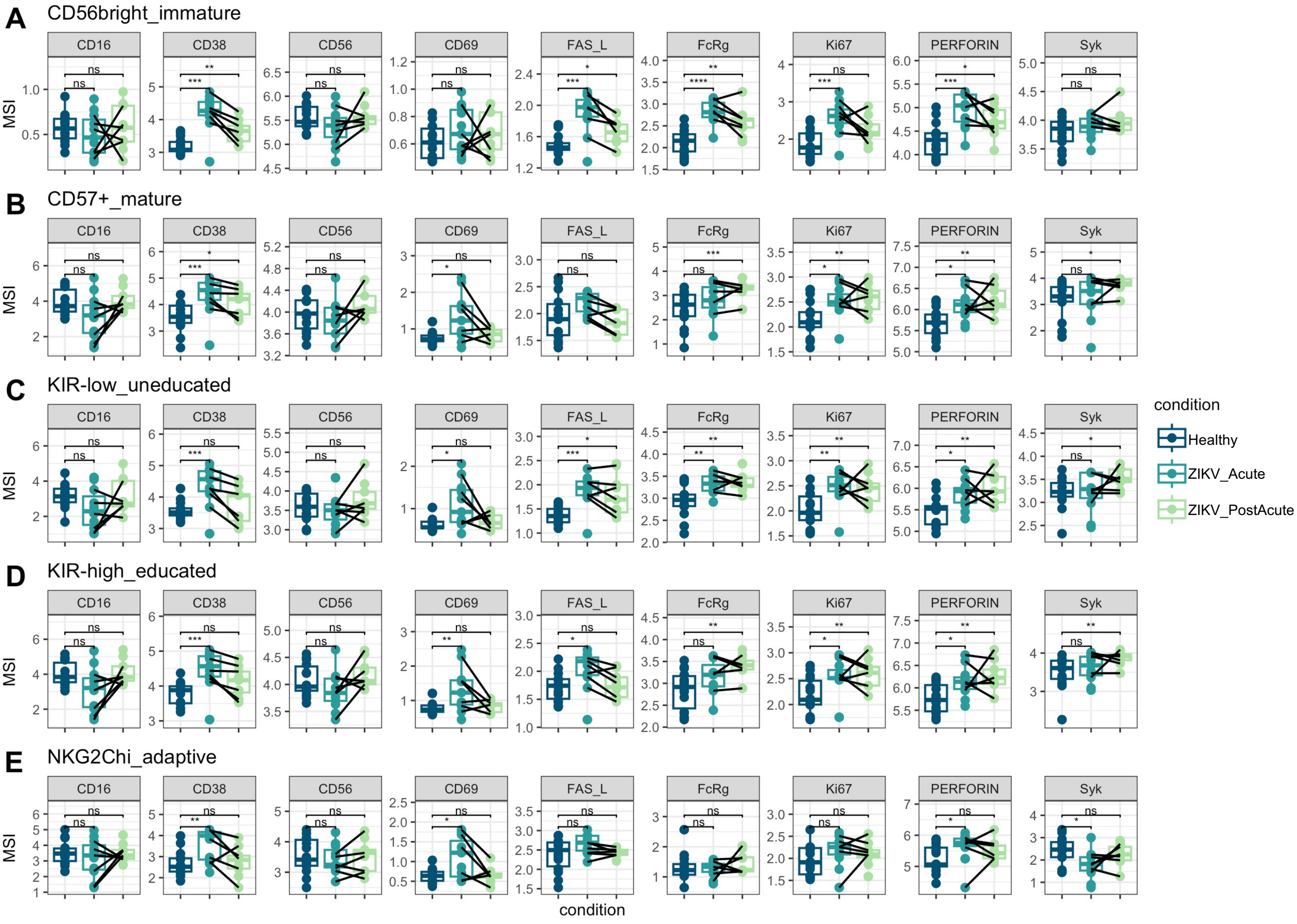
A CD56bright_immature
B CD57+_mature
C KIR-low_uneducated
D KIR-high_educated
E NKG2Chi_adaptive
CD16
CD38
CD56
CD69
FAS_L
FcRg
Ki67
PERFORIN
Syk
MSI
condition
Healthy
ZIKV_Acute
ZIKV_PostAcute

Figure S4

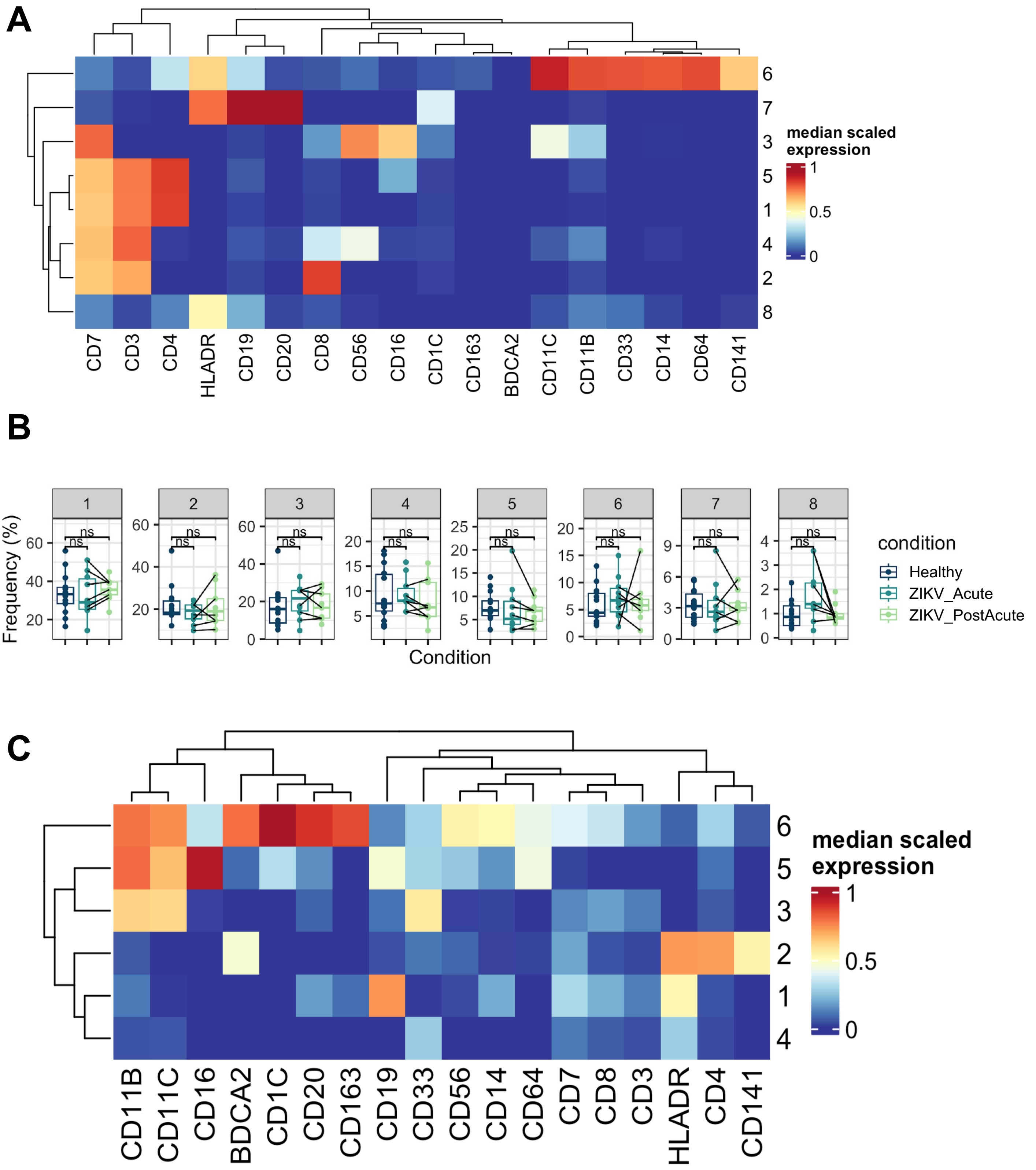
A
median scaled expression
1
0.5
0
6
7
3
5
1
4
2
8
CD7
CD3
CD4
HLADR
CD19
CD20
CD8
CD56
CD16
CD1C
CD163
BDCA2
CD11C
CD11B
CD33
CD14
CD64
CD141
B
1
2
3
4
5
6
7
8
ns
Frequency (%)
Condition
condition
Healthy
ZIKV_Acute
ZIKV_PostAcute
C
median scaled expression
1
0.5
0
6
5
3
2
1
4
CD11B
CD11C
CD16
BDCA2
CD1C
CD20
CD163
CD19
CD33
CD56
CD14
CD64
CD7
CD8
CD3
HLADR
CD4
CD141

Figure S5

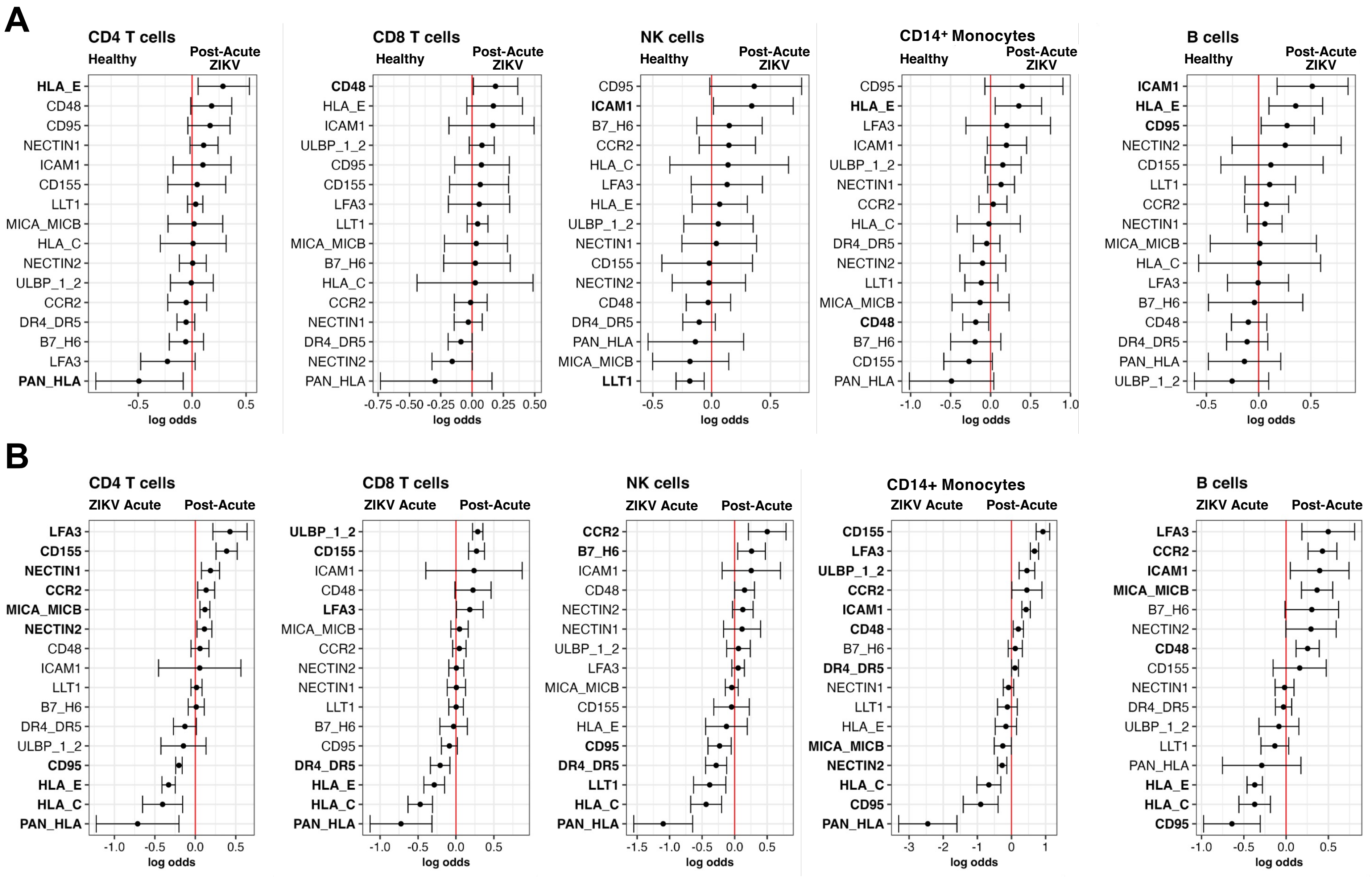

A
CD4 T cells
Healthy
Post-Acute ZIKV
HLA_E
CD48
CD95
NECTIN1
ICAM1
CD155
LLT1
MICA_MICB
HLA_C
NECTIN2
ULBP_1_2
CCR2
DR4_DR5
B7_H6
LFA3
PAN_HLA
-0.5
0.0
0.5
log odds
CD8 T cells
Healthy
Post-Acute ZIKV
CD48
HLA_E
ICAM1
ULBP_1_2
CD95
CD155
LFA3
LLT1
MICA_MICB
B7_H6
HLA_C
CCR2
NECTIN1
DR4_DR5
NECTIN2
PAN_HLA
-0.75 -0.50 -0.25 0.00 0.25 0.50
log odds
NK cells
Healthy
Post-Acute ZIKV
CD95
ICAM1
B7_H6
CCR2
HLA_C
LFA3
HLA_E
ULBP_1_2
NECTIN1
CD155
NECTIN2
CD48
DR4_DR5
PAN_HLA
MICA_MICB
LLT1
-0.5
0.0
0.5
log odds
CD14+ Monocytes
Healthy
Post-Acute ZIKV
CD95
HLA_E
LFA3
ICAM1
ULBP_1_2
NECTIN1
CCR2
HLA_C
DR4_DR5
NECTIN2
LLT1
MICA_MICB
CD48
B7_H6
CD155
PAN_HLA
-1.0
-0.5
0.0
0.5
1.0
log odds
B cells
Healthy
Post-Acute ZIKV
ICAM1
HLA_E
CD95
NECTIN2
CD155
LLT1
CCR2
NECTIN1
MICA_MICB
HLA_C
LFA3
B7_H6
CD48
DR4_DR5
PAN_HLA
ULBP_1_2
-0.5
0.0
0.5
log odds
B
CD4 T cells
ZIKV Acute
Post-Acute
LFA3
CD155
NECTIN1
CCR2
MICA_MICB
NECTIN2
CD48
ICAM1
LLT1
B7_H6
DR4_DR5
ULBP_1_2
CD95
HLA_E
HLA_C
PAN_HLA
-1.0
-0.5
0.0
0.5
log odds
CD8 T cells
ZIKV Acute
Post-Acute
ULBP_1_2
CD155
ICAM1
CD48
LFA3
MICA_MICB
CCR2
NECTIN2
NECTIN1
LLT1
B7_H6
CD95
DR4_DR5
HLA_E
HLA_C
PAN_HLA
-1.0
-0.5
0.0
0.5
log odds
NK cells
ZIKV Acute
Post-Acute
CCR2
B7_H6
ICAM1
CD48
NECTIN2
NECTIN1
ULBP_1_2
LFA3
MICA_MICB
CD155
HLA_E
CD95
DR4_DR5
LLT1
HLA_C
PAN_HLA
-1.5
-1.0
-0.5
0.0
0.5
log odds
CD14+ Monocytes
ZIKV Acute
Post-Acute
CD155
LFA3
ULBP_1_2
CCR2
ICAM1
CD48
B7_H6
DR4_DR5
NECTIN1
LLT1
HLA_E
MICA_MICB
NECTIN2
HLA_C
CD95
PAN_HLA
-3
-2
-1
0
1
log odds
B cells
ZIKV Acute
Post-Acute
LFA3
CCR2
ICAM1
MICA_MICB
B7_H6
NECTIN2
CD48
CD155
NECTIN1
DR4_DR5
ULBP_1_2
LLT1
PAN_HLA
HLA_E
HLA_C
CD95
-1.0
-0.5
0.0
0.5
log odds

Figure S6

A

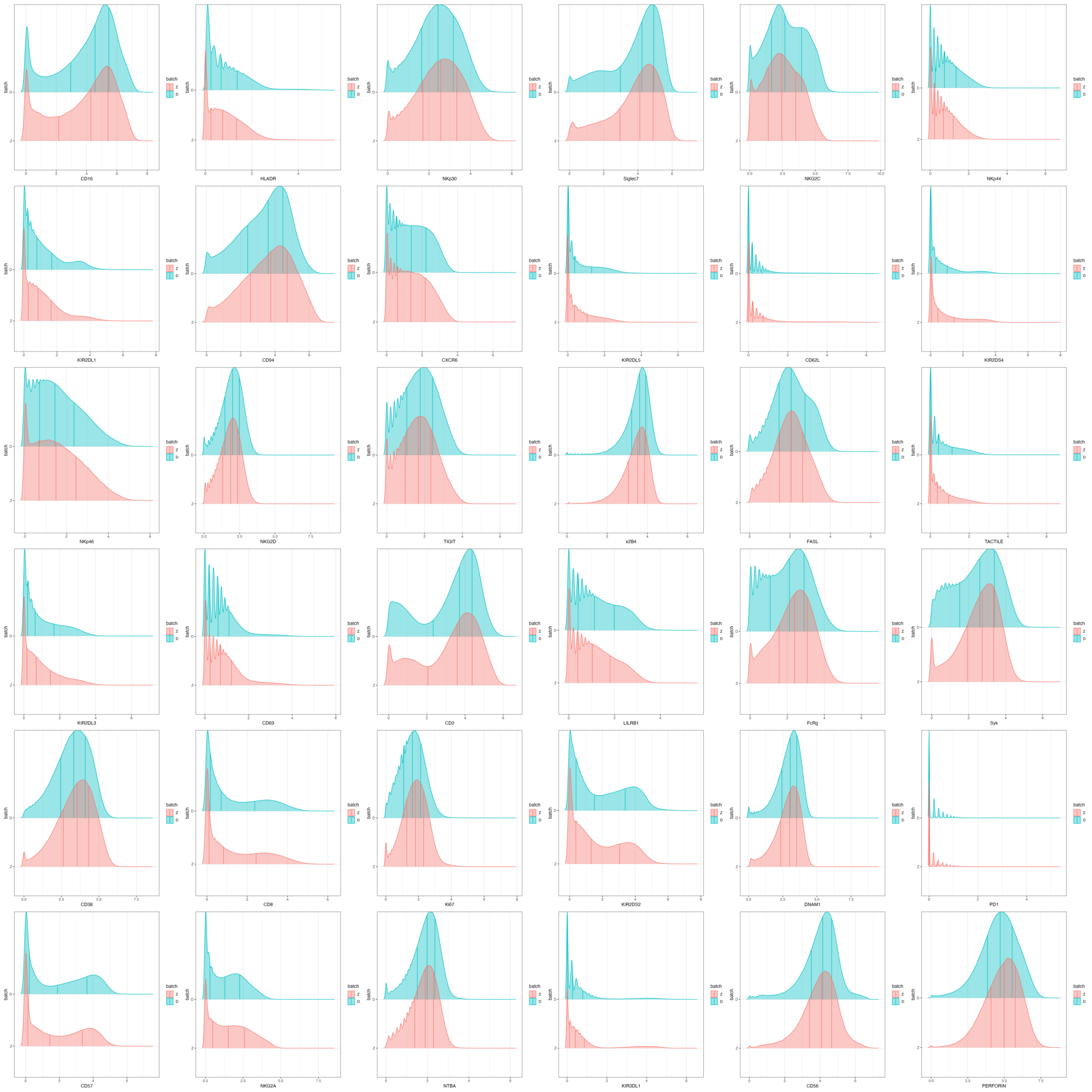

B

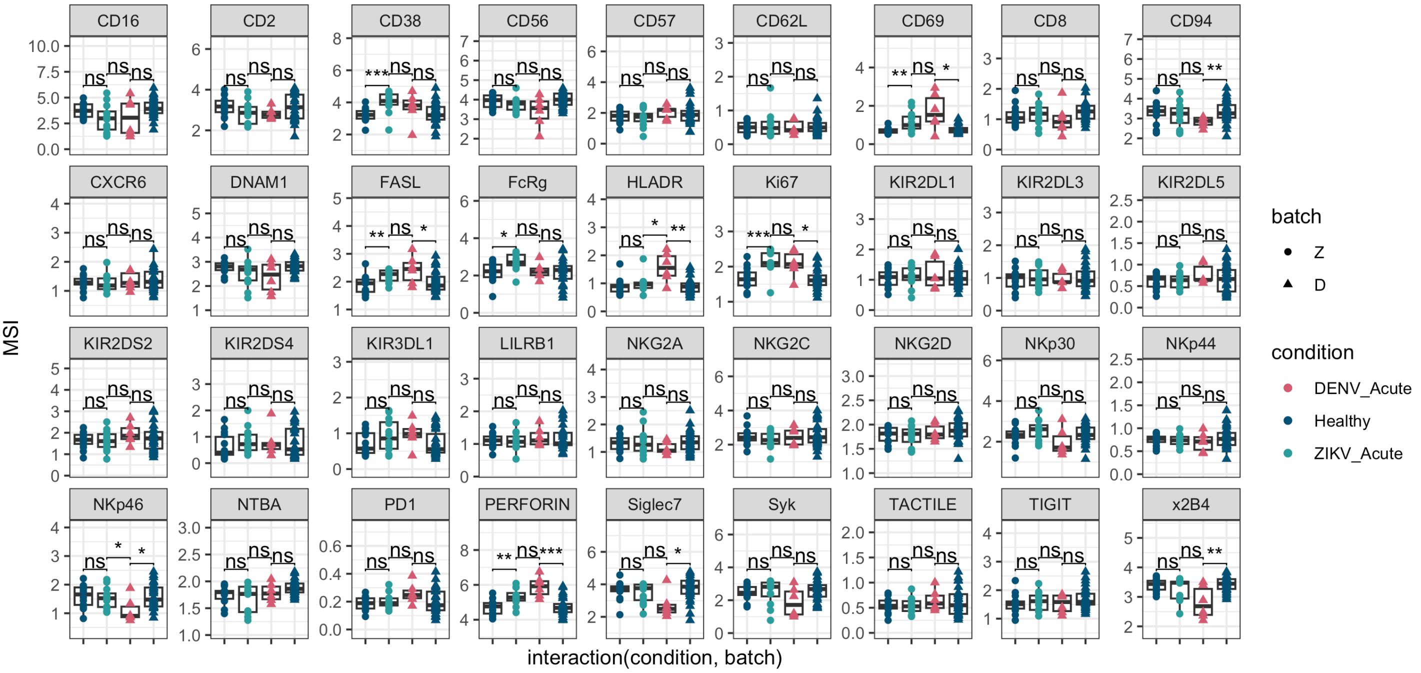

CD16
CD2
CD38
CD56
CD57
CD62L
CD69
CD8
CD94
CXCR6
DNAM1
FASL
FcRg
HLADR
Ki67
KIR2DL1
KIR2DL3
KIR2DL5
KIR2DS2
KIR2DS4
KIR3DL1
LILRB1
NKG2A
NKG2C
NKG2D
NKp30
NKp44
NKp46
NTBA
PD1
PERFORIN
Siglec7
Syk
TACTILE
TIGIT
x2B4
MSI
interaction(condition, batch)
batch
Z
D
condition
DENV_Acute
Healthy
ZIKV_Acute

bioRxiv preprint doi: https://doi.org/10.1101/2025.09.15.676203; this version posted September 16, 2025. The copyright holder for this preprint (which was not certified by peer review) is the author/funder, who has granted bioRxiv a license to display the preprint in perpetuity. It is made available under aCC-BY 4.0 International license.

Table S1: NK Ligand CyTOF panel. Antibodies in red were spiked into the frozen panel separately

Isotope	Protein	Vendor	Clone	Note
89Y	HLA-DR	Biolegend	L243	surface
112Cd	CD19 Qdot	Life Technologies	SJ25-C1	surface
115In	CD3	Biolegend	UCHT1	surface
141Pr	CD20	Biolegend	2H7	surface
142Nd	CD163	Biolegend	GHI/61	surface
143Nd	PAN HLA	Biolegend	W6/32	surface
144Nd	CD7	Biolegend	CD7-6B7	surface
145Nd	CD8	Biolegend	SK1	surface
146Nd	CD48	Biolegend	BJ40	surface
147Sm	BDCA-2	Biolegend	201A	surface
148Nd	ICAM1 (CD54)	Biolegend	HA58	surface
149Sm	LLT-1	R&D	402659	surface
151Eu	CD4	Biolegend	OKT4	surface
152Sm	CD64	Biolegend	10.1	surface
153Eu	HLA-C	Millipore	DT9	surface
154Sm	CCR2	Biolegend	K036C2	surface
155Gd	HLA-E	Biolegend	3D12	surface
156Gd	CD95	Biolegend	DX2	surface
157Gd	Nectin-1 (CD111)	Biolegend	R1.302	surface
158Gd	MICA/MICB	R&D	159227/236511	surface
159Tb	DR4 (CD261)	Biolegend	DJR1	surface
159Tb	DR5 (CD262)	Biolegend	DJR2-2	surface
160Gd	CD1c	Biolegend	L161	surface
161Dy	ULBP-1/2 (5 and 6)	R&D	170818/165903	surface
162Dy	CD11c	Biolegend	Bu15	surface
164Dy	Nectin-2 (CD112)	Biolegend	TX31	surface
165Ho	CD155 (PVR)	Biolegend	SKII.4	surface
166Er	HLA-Bw4	Miltenyi	REA274	surface
167Er	CD32	STEMCELL	IV.3	surface
168Er	HLA-Bw6	Miltenyi	REA143/ 130-095-212	surface
169Tm	CD14	Biolegend	M5E2	surface
170Er	CD11b	Biolegend	ICRF44	surface
171Yb	LFA-3 (CD58)	Biolegend	TS2/9	surface
172Yb	CD33	Biolegend	WM53	surface
173Yb	CD141	Fluidigm pre-conjugated	1A4	surface
174Yb	CD56	BD Pharmingen	NCAM16.2	surface
175Lu	CD86	Biolegend	IT2.2	surface
176Yb	B7-H6	R&D	875001	surface
209Bi	CD16	Fluidigm pre-conjugated	3G8	surface
150Nd	Flavi E-protein	EMD Millipore	D1-4G2-4-15	intracellular

bioRxiv preprint doi: https://doi.org/10.1101/2025.09.15.676203; this version posted September 16, 2025. The copyright holder for this preprint (which was not certified by peer review) is the author/funder, who has granted bioRxiv a license to display the preprint in perpetuity. It is made available under aCC-BY 4.0 International license.

Table S2: NK cell CyTOF panel. Antibodies in red were spiked into the lyophilized panel separately.

Isotope	Marker	Source	Clone	Notes
89Y	CD57	Biolegend	HCD57	Surface
Qdot	HLA-DR	Life technologies	Tu36	Surface
115In	CD3	Biolegend	UCHT	Surface
141Pr	CD38	Biolegend	HIT2	Surface
142Nd	CD69	Biolegend	FN50	Surface
143Nd	CD33	Biolegend	WM53	Surface
143Nd	CD14	Biolegend	M5E5	Surface
144Nd	CD2	Biolegend	RPA-2.10	Surface
145Nd	LILRB1	Biolegend	GHI/75	Surface
146Nd	CD19	Biolegend	HIB19	Surface
147Sm	CD8	Biolegend	SK1	Surface
149Sm	CD4	Biolegend	SK3	Surface
151Eu	CD62L	Biolegend	DREG-56	Surface
153Eu	KIR2DS4	R&D systems	179315	Surface
154Sm	KIR2DS2	Abcam	Poly-clonal	Surface
155Gd	NKp46	Biolegend	9E2	Surface
156Gd	NKG2D	Biolegend	1D11	Surface
157Gd	TIGIT	R&D systems	741182	Surface
158Gd	2B4	Biolegend	C1.7	Surface
159Tb	DNAM-1	BD biosciences	DX11	Surface
160Gd	FAS-L	Biolegend	NOK-1	Surface
161Dy	NKp30	Biolegend	P30-15	Surface
162Dy	Siglec-7	Biolegend	S7.7	Surface
163Dy	NKG2C	R&D systems	134522	Surface
164Dy	NKp44	Biolegend	P44-8	Surface
165Ho	TACTILE	Biolegend	NK92.39	Surface
166Er	KIR2DL1	R&D systems	143211	Surface
167Er	CD94	Biolegend	DX22	Surface
168Er	CXCR6	Biolegend	K041E5	Surface
169Tm	PD1	Biolegend	EH12.2H7	Surface
170Er	KIR2DL5	Miltenyi	UP-R1	Surface
171Yb	NKG2A	R&D systems	131411	Surface
172Yb	NTB-A	Biolegend	NT-7	Surface
173Yb	KIR3DL1	BD biosciences	DX-9	Surface
174Yb	CD56	BD biosciences	NCAM16.2	Surface
175Lu	KIR2DL3	R&D systems	180701	Surface
209Bi	CD16	Fluidigm	3G8	Surface
148Nd	FcRg	Millipore	polyclonal	intracellular
150Nd	Syk	Biolegend	4D10.2	intracellular
152Sm	Ki-67	Biolegend	Ki-67	intracellular
176Yb	Perforin	Abcam	B-D48	intracellular
